# Anti-Parstatin Promotes Angiogenesis and Ameliorates Left Ventricular Dysfunction during Pressure Overload

**Published:** 2014-03

**Authors:** Srikanth Givvimani, Nithya Narayanan, Sathnur Basappa Pushpakumar, Suresh C. Tyagi

**Affiliations:** Department of Physiology and Biophysics, University of Louisville School of Medicine, Louisville, KY-40202, USA

**Keywords:** Aortic banding, Matrix remodeling, Angiogenesis, Anti-angiogenic factors, Heart failure

## Abstract

Parstatin, a novel protease activated receptor-1 (PAR-1) derived peptide is a potent inhibitor of angiogenesis. We and others have reported that imbalance between angiogenic growth factors and anti-angiogenic factors results in transition from compensatory cardiac hypertrophy to heart failure in a pressure overload condition. Though cardio protective role of parstatin was shown previously in ischemic cardiac injury, its role in pressure overload cardiac injury is yet to unveil. We hypothesize that supplementing anti-parstatin antibody during pressure overload condition augments angiogenesis and ameliorate left ventricular dysfunction and heart failure. To verify this, we created ascending aortic banding in mice to mimic pressure overload condition and then treated mice with anti-parstatin antibody. Left ventricular function was assessed by echocardiography and pressure-volume loop study. Angiogenic growth factors and anti-angiogenic factors along with MMP-2,-9 were evaluated by western blot and immunohistochemistry. Results: our results showed an improved left ventricular function in anti-parstatin treated aortic banding hearts compared to their corresponding wild type controls. Expression of angiogenic growth factor, VEGF, MMP-2 and CD31 expression was increased in treated aortic banding hearts compared to their corresponding wild type controls. Our results suggest that treating pressure overload mice with anti-parstatin antibody augments angiogenesis and ameliorates left ventricular dysfunction.

## INTRODUCTION

We and others have previously reported the importance of angiogenesis during transition from compensatory cardiac hypertrophy to de-compensatory heart failure in a pressure overload mice models (1-3) . Angiogenesis depends on the balance between the factors that stimulate or impede angiogenesis (4). Vascular endothelial growth actor (VEGF) is a well identified and validated potent angiogenic growth factor (5). Of late anti-angiogenic factors are also emerging as therapeutic targets (6, 7) . Some of the anti-angiogenic factors are angiostatin, endostatin and parstatin. Although, we have shown that during pressure overload condition, expression of angiostatin and endostatin was increased in de-compensatory stage (1, 2, 8) , effect of parstatin (anti-angiogenic factor) inhibition during the transition from compensatory cardiac hypertrophy to failure is unknown. Thus we hypothesize that supplementing anti-parstatin antibody during pressure overload condition augments angiogenesis and ameliorates left ventricular dysfunction and heart failure. Parstatin is a cleaved peptide of proteinase activated receptor-1 (PAR-1), released during its activation by thrombin (9) . PAR-1 is a G protein coupled receptor (GPCR) that mediates the angiogenic activity of thrombin (9-11) . Interestingly parstatin regulates PAR-1 receptor and is a potent inhibitor of angiogenesis (9) . The growth inhibitory effect of parstatin was shown in specific to bFGF (fibroblast growth factor) and VEGF induced angiogenesis (11) . Exogenous administration of parstatin compound was shown to confer cardio protection in various ischemia reperfusion studies (12, 13) . It was reported that in both *in vivo* and *in vitro* model of angiogenesis, parstatin inhibits endothelial cell proliferation and angiogenesis (9, 14) . Parstatin was also reported as pro-apoptotic compound and leads to caspase dependent activation of programmed cell death (9) . Current study details the role of anti-parstatin in improving the angiogenesis in pressure overload heart and ameliorating the left ventricular dysfunction.

## MATERIALS AND METHODS

### Animals

Wild type mice (WT, C57BL6/J) aged 8 weeks were procured from Jackson Laboratories (Bar Harbor, Me.; USA) and housed in the animal care facility at University of Louisville with access to standard chow and water. Ascending aortic banding was created in mice of 12 weeks age with an approximate weight of 23-25 grams. After the study period animals were euthanized in accordance with National Institute of Health Guidelines for animal research and were reviewed and approved by the Institute Animal Care and use Committee of University of Louisville (IACUC # 07134).

### Pressure overload animal model

Ascending aortic banding was done as described previously (1, 2). Briefly, under sodium pentobarbital anesthesia, animals were intubated and ventilated with Harvard mini ventilator. Body temperature was maintained with a heating pad. Under sterile surgical environment thorax was opened by left parasternal thoracotomy and ascending aorta was dissected and separated from the adjacent structures. Ascending aorta was ligated with 6-0 silk by placing the 26 g needle on the aorta for optimum constriction. Needle was quickly removed to keep the constricted aorta patent. Wound was closed in layers using 6-0 vicryl for the subcutaneous tissues and 5-0 silk to the skin. The mortality rate was less than 20% with the surgery. All animals were given post-operative analgesia with intraperitoneal injection of Ketofen, 5 mg/Kg body weight. Sham group of animals underwent similar procedure except the aortic constriction. By creating pressure overload using ascending aortic banding model, heart failure develops by 8 weeks. Our previous experiments showed the pathophysiological and histological changes associated with heart failure are achieved by 8 weeks (1, 2, 8).

### Anti-parstatin treatment

Custom manufactured antibody to mouse parstatin peptide was ordered from EZ Biolab, Carmel, IN, USA. Anti parstatin antibody was administered as intra peritoneal injection at a dose of 50 μg/Kg body weight on alternative days for 5 weeks. Our preliminary results didn’t show any significant effect of anti-parstatin on sham group of mice. At the end of the treatment, animals were euthanized and organs were harvested and stored at -80°C.

### Antibodies & Reagents

The following primary antibodies were used for immunohistochemical data: mouse polyclonal MMP-2, rabbit polyclonal MMP-9 antibodies were purchased from Abcam (Cambridge, MA). Anti-mouse VEGF antibody was purchased from R&D systems (Minneapolis, MN). Anti-mouse CD 31 or PECAM (Platelet endothelial cell adhesion molecule) was purchased from BD Pharmingen (Sandiego, CA). Cleaved caspace-3 antibody (Cell Signaling #9661) from rabbit source was used. Following fluorescent secondary antibodies for Immunohistochemistry (IHC) were ordered from Invitrogen (Carlsbad, CA): Texas Red raised in mouse, Alexa Fluor 488, 594 raised in rabbit and Alexa fluor 647 raised in rat.

### Echocardiography

Left ventricular functional status was assessed by transthoracic echocardiography as described elsewhere (1) . Briefly, echo was performed on mice to achieve two dimensional left ventricle images from an apical view using a SONOS 5500 or 2500; Hewlett-Packard, Inc. and a 12.5 MHz transducer. Tribromo ethanol (TBE) anesthesia (intra peritoneal dose of 240 mg/kg body weight), was used to minimize the cardio depressing action. Mice were depilated with hair removal cream (Nair) and placed on a heating pad to maintain body temperature. The functional status of the heart was assessed by LVIDd, LVIDs, LVPWD and %FS. %FS is the most common parameter used to evaluate left ventricular function in murine echocardiography (15).

### Cryosectioning

After euthanizing the mice, heart tissue was harvested and washed thoroughly in phosphate buffered saline (PBS) and cryo-preserved with liquid nitrogen in a Peel-A-Way disposable plastic tissue embedding moulds (Polysciences inc., Warrington, PA.,USA) having tissue freezing media (Triangle Biomedical Sciences, Durham, N.C., USA) and stored at -70°C until further use. 5 μm thickness tissue sections were made using Cryocut (Leica CM 1850) and placed on Super frost plus microscope slides, air-dried and processed for staining.

### Immunohistochemistry

5 mm thick frozen sections of the heart were used to perform immunohistochemistry (IHC) following standard IHC protocol (Abcam) as described previously. Following overnight primary antibody application, secondary antibodies were applied for 2 hours at room temperature and stained slides were mounted and visualized with fluorescence by a laser scanning confocal microscope (Olympus FluoView1000) with an appropriate filter.

### Masson’s Trichrome Staining

Fibrosis or collagen deposition in frozen tissue sections was assessed by Mason’s trichrome (Richard-Allan Scientific, Kalamazoo, MI, USA) staining following the manufacturer’s instructions. Collagen appeared as blue color staining.

### Image Proplus software

Images from Immunohistochemistry and Masson’s trichrome staining were analyzed with Image Proplus software. Image analysis was done using image-pro software in confocal images taking different optical fields at random into consideration while the densitometry analysis was done using Bio-Rad software.

### Statistical analysis

All data are expressed as mean ± SE. One-way analysis of variance (ANOVA) was performed to test for treatment effects, and differences between groups were determined using Tukey’s post-hoc test. We used the primer of Biostatistics software to analyze our data. A *p* value <0.05 was considered to be significant.

## RESULTS

### Treatment with anti-parstatin improves left ventricular dysfunction

**Figure 1:**
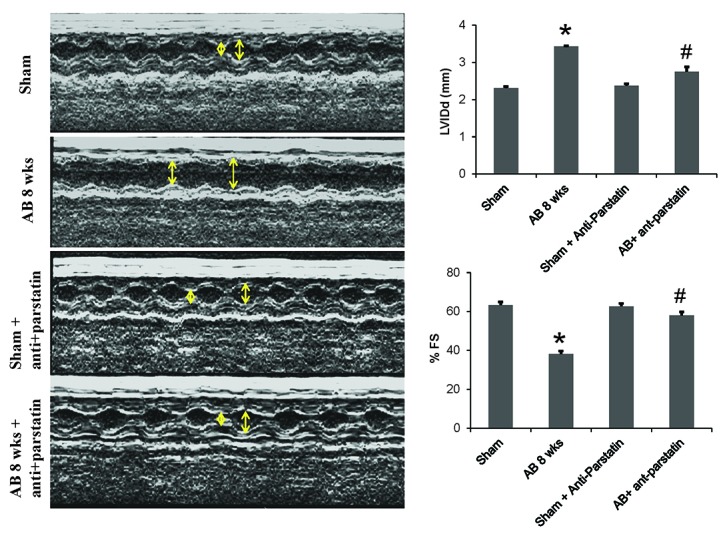
M-mode echocardiography images depicting the left ventricular chamber (LVID) and posterior wall dimension. Left ventricular function assessed by % fractional shortening (%FS) and LVIDd is represented in adjacent bar diagram. *p<0.05 compared to sham and #*p<*0.05 compared to AB 8 wks group.

Left ventricular function assessed by echocardiography revealed that the administration of anti-parstatin antibody in a pressure overload mice models improves ventricular dysfunction compared to the controls. M-mode echo showed decreased ventricular chamber diameter in treated group of mice. Left ventricular functional parameter, percentage fractional shortening (%FS) was also significantly improved in anti-parstatin treated group compared to the corresponding controls. Administration of anti-parstatin in sham group didn’t show any changes (Figure 1).

### Anti-parstatin promotes angiogenesis

**Figure 2:**
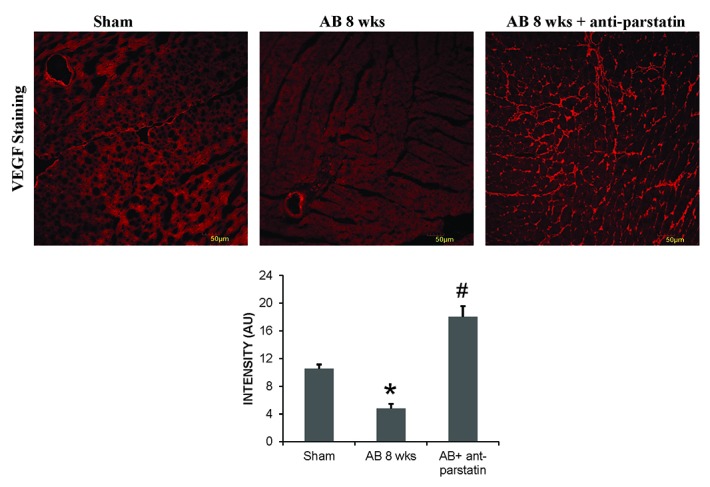
IHC staining of frozen heart sections with VEGF antibody. Quantified data from the images was represented in the adjacent bar graphs. *p<0.05 compared to sham and #*p<*0.05 compared to AB 8 wks group.

**Figure 3:**
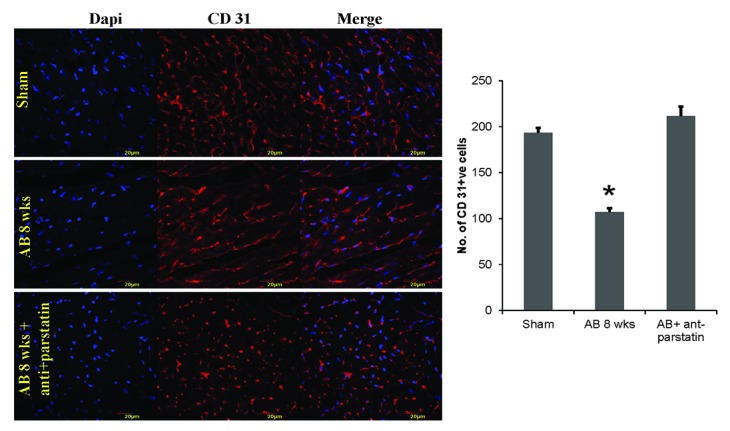
IHC staining for CD 31 to assess endothelial capillary density. Red fluorescence depicts CD 31 and dapi blue for nuclei. Quantified data from the images is represented in the adjacent bar graphs. *p<0.05 compared to sham.

Parstatin is a well reported potent angiogenic inhibitor. Treatment with anti-parstatin in a pressure overload mice models of hypertension promotes endothelial growth and angiogenesis. Expression of VEGF, a potent angiogenic marker was significantly increased in anti-parstatin treated mice group. Also, platelet endothelial cell adhesion marker, CD 31 expression was also increased in the treatment group suggesting neo-angiogenesis (Figure 2 and Figure 3).

### Anti-parstatin regulates MMP-9 but promotes MMP-2 expression

**Figure 4:**
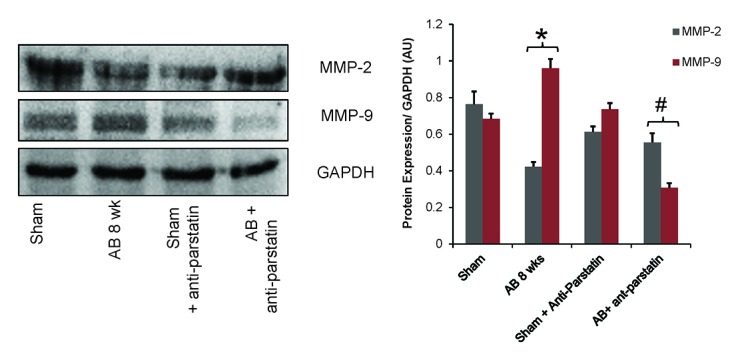
Western blot data showing protein expression of MMP-9 and MMP-2 along with GAPDH. Quantified densitometry units normalized with GAPDH are represented in adjacent bar graphs. *p<0.05 compared to sham and #*p<*0.05 compared to AB 8 wks group.

**Figure 5:**
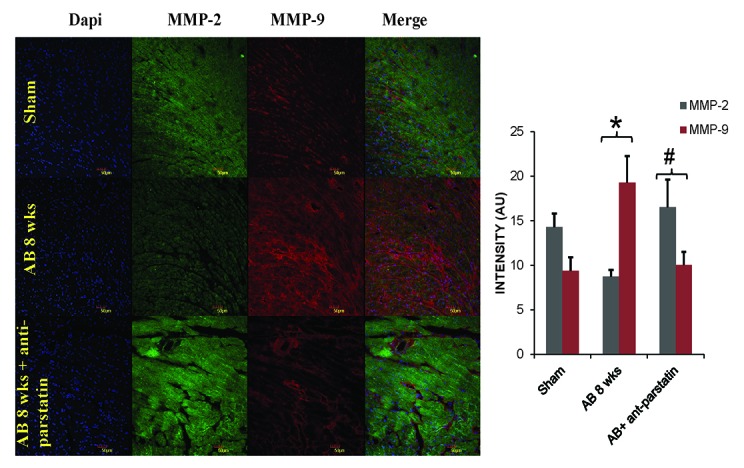
IHC staining of frozen heart sections with MMP-2 (green fluorescence) and MMP-9 (red fluorescence). Quantified fluorescence intensity was depicted in adjacent bar graphs. *p<0.05 compared to sham and #*p<*0.05 compared to AB 8 wks group.

Matrix metalloproteinases play an important role in angiogenesis by promoting either pro-angiogenic growth factors or anti-angiogenic factors. We have shown previously that during compensatory cardiac hypertrophy, MMP-2 expression was increased promoting angiogenesis while MMP-9 expression supersedes in de-compensatory heart failure promoting anti-angiogenesis. Current findings reveal that the treatment with anti-parstatin promotes pro-angiogenic MMP-2 expression but reduces MMP-9 expression suggesting improved angiogenesis (Figure 4 and Figure 5). Anti-parstatin treatment in sham group of mice didn’t elicit any major significant changes compared to sham control.

### Expression of caspase was decreased in anti-parstatin treatment group

**Figure 6:**
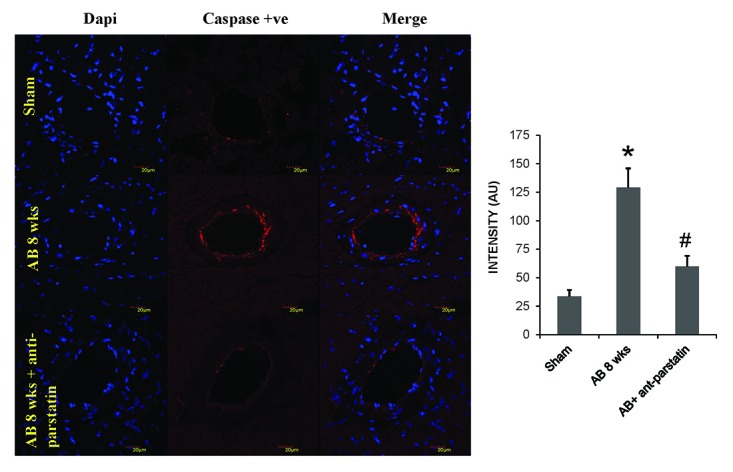
IHC staining for caspase -3 to assess apoptotic activity. Red fluorescence depicts caspase activity and dapi blue for nuclei. Quantified data from the images is represented in the adjacent bar graphs. *p<0.05 compared to sham and #*p<*0.05 compared to AB 8 wks group.

**Figure 7:**
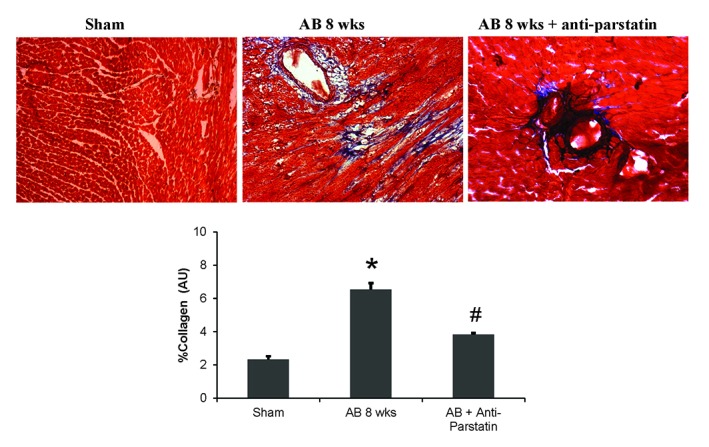
Masson’s trichrome staining depicting collagen deposition in heart tissue sections of mice. Quantified percentage of collagen is represented in bar diagram. *p<0.05 compared to WT control and #*p<*0.05 compared to AB 8 wks group.

Parstatin was shown to promote apoptosis and this mechanism is mediated by the activation of caspase enzyme. Our findings in the current study showed that caspase expression was decreased in treatment group compared to control group suggesting decreased apoptosis (Figure 6). Also, collagen expression analyzed by Masons trichrome staining showed decreased collagen deposition or fibrosis in the treatment group compared to untreated control group (Figure 7).

## DISCUSSION

In the present study we tried to explain the protective effects of anti-parstatin in pressure overload condition. We have shown previously that transition from compensatory cardiac hypertrophy to de-compensatory heart failure is mainly due to decreased angiogenesis due to increased anti-angiogenic factors like angiostatin, endostatin and parstatin (1, 2, 16) . When anti-parstatin is administered in post aortic banding mice, it promotes angiogenesis and mitigates left ventricular dysfunction. Parstatin was shown as potential endogenous angiogenesis inhibitor in previous studies (9) . Parstatin is a cleaved peptide of PAR-1 during activation of thrombin. Earlier reports show the evidence that parstatin may be released from thrombin activated platelets (17, 18) . Association of PAR-1 to angiogenesis has been well reported in previous literature (19, 20) . PAR-1 levels were shown to be up-regulated during the process of wound healing, cancer, inflammation and repair, where angiogenesis is initiated (19-22) . We and others have reported the role of protease activated receptors in various cardiovascular diseases (23-25) . Though PAR-1 levels are associated with angiogenesis, it is interesting that its cleaved peptide, parstatin is a potent anti-angiogenic factor. It was reported that inhibition effect of parstatin is mainly against VEGF and bFGF angiogenic factors.

**Figure 8:**
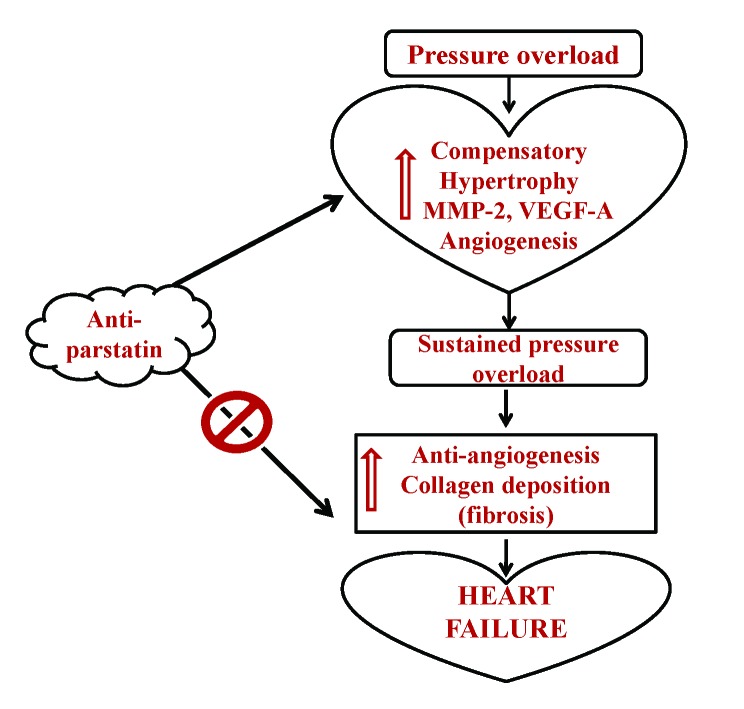
Schematic of hypothesis.

On the other hand, cardio-protective role of parstatin was reported in various ischemia and reperfusion studies (12, 13) . Recently it was reported that parstatin is also renal protective in ischemia reperfusion injury and radio contrast injury to kidneys (26) . It is evident that reperfusion injury in an ischemia setting is mainly related to angiogenesis and thus in these situations protective role of parstatin is mainly through inhibition of angiogenesis. In contrast, in a pressure overload setting where angiogenesis is required to prevent the transition of compensatory hypertrophy to heart failure, inhibiting parstatin would be cardio protective. Our results showed that by administering anti-parstatin antibody following pressure overload aortic banding ameliorates left ventricular dysfunction by increasing the pro-angiogenic VEGF and capillary endothelial marker CD31 (Figure 2 and Figure 3). Our previous studies have shown the role of matrix metalloproteinases induced angiogenesis in pressure overload aortic banding injury. We have reported that MMP-2 is pro-angiogenic and MMP-9 is anti angiogenic (1, 2, 8) . Findings from the present study shows that anti parstatin antibody increases MMP-2 expression but decreases MMP-9 expression (Figure 4 and Figure 5). It was reported that parstatin is a proapoptotic compound and the apoptotic activity is mediated by caspase activation. It was also shown that this proapoptotic activity of parstatin was reversed by a caspase inhibitor (9) . We have shown previously that during pressure overload aortic banding there is significant increase in caspase activity and apoptotic cell death that leads to fibrosis (8) . Similarly in our current study we found that anti-parstatin antibody supplementation decreased caspase activity and cardiac collagen deposition or fibrosis (Figure 6 and Figure 7) suggesting decreases apoptosis. We are the first to report the novel findings of cardio protective anti parstatin antibody in a pressure overload mice models. We found that anti parstatin antibody ameliorates left ventricular dysfunction by increasing the angiogenesis and decreasing the caspase activity and cardiac fibrosis (Figure 8).

## CONCLUSIONS

Our results demonstrate that anti-parstatin administration in pressure overload mice increases angiogenesis. Anti-parstatin also inhibits caspase mediated activation of apoptosis and decreases collagen deposition during pressure overload condition. We conclude that treating pressure overload mice with anti-parstatin augments angiogenesis and ameliorates left ventricular dysfunction.
